# Why patients want to take or refuse to take antibiotics: an inventory of motives

**DOI:** 10.1186/s12889-019-6834-x

**Published:** 2019-04-27

**Authors:** Adriana Bagnulo, Maria-Teresa Muñoz Sastre, Lonzozou Kpanake, Paul Clay Sorum, Etienne Mullet

**Affiliations:** 1 Jean-Jaurès University, CERPPS, Maison de la recherche, 5 allées Antonio Machado, 31058 Toulouse cedex 9, France; 2 University of Québec (TELUQ), 5800, rue Saint-Denis, Bureau 1105, Montréal, Québec H2S 3L5 Canada; 3 Albany Medical College, Albany, Latham Med-Ped, 724 Watervliet-Shaker Road, Latham, NY 12110 USA; 4 Institute of Advanced Studies (EPHE), 17 bis, rue Quefes, Plaisance du Touch, 31830 Paris, France

**Keywords:** Antibiotics, Adherence to treatment, Non-adherence, Patients’ preferences

## Abstract

**Background:**

Inappropriate use of antibiotics is a worldwide issue. In order to help public health institutions and each particular physician to change patterns of consumption among patients, it is important to understand better the reasons why people accept to take or refuse to take the antibiotic drugs. This study explored the motives people give for taking or refusing to take antibiotics.

**Methods:**

Four hundred eighteen adults filled out a 60-item questionnaire that consisted of assertions referring to reasons for which the person had taken antibiotics in the past and a 70-item questionnaire that listed reasons for which the person had sometimes refused to take antibiotics.

**Results:**

A six-factor structure of motives to take antibiotics was found: *Appropriate Prescription, Protective Device, Enjoyment* (antibiotics as a quick fix allowing someone to go out)*, Others’ Pressure, Work Imperative,* and *Personal Autonomy.* A four-factor structure of motives not to take antibiotics was found: *Secondary Gain* (through prolonged illness)*, Bacterial Resistance, Self-defense* (the body is able to defend itself) and *Lack of trust.* Scores on these factors were related to participants’ demographics and previous experience with antibiotics.

**Conclusion:**

Although people are generally willing to follow their physician’s prescription of antibiotics, a notable proportion of them report adopting behaviors that are beneficial to micro-organisms and, as a result, potentially detrimental to humans.

**Electronic supplementary material:**

The online version of this article (10.1186/s12889-019-6834-x) contains supplementary material, which is available to authorized users.

## Background

Antibiotics consumption is on the rise in most countries, especially in countries forming the BRICS (Brazil, Russia, India, China and South-Africa) group [1]. Inappropriate use of antibiotics is a worldwide issue that concerns developed countries as well as developing countries. This issue can, nevertheless, arise differently from one part of the world to another, depending on the level of economic development and local cultures [2]. Irresponsible antibiotic use may have detrimental effects—increasing antibiotic resistance (the public health level), and causing side effects (the patient level), either directly through gastrointestinal side effects and allergic reactions or indirectly by changing the nature of the gut flora [3, 4]. In order to help public institutions and each particular physician to change patterns of consumption among patients, it is important to understand better the reasons why people (a) accept to take antibiotics when they are prescribed, (b) self-medicate themselves when denied the expected prescription, or (c) refuse to take the antibiotic drugs that have been duly prescribed [5].

Prescription by a physician is certainly not the only reason to take antibiotics: Patients consume many over-the-counter pharmacological substances, and antibiotics are just one of them. In France, antibiotics are available only on a physician’s prescription, but they may be borrowed from family members or they can be bought on the Internet. The reasons for antibiotic use by patients have, until now, not been examined in a systematic way. Studies about patients’ expectations regarding antibiotic prescription converge on the view that physicians tend to overestimate patients’ expectations [6, 7]. Studies about patients’ level of knowledge about antibiotic use converge on the view that it is quite poor, and, in particular, that the erroneous belief that antibiotics are indicated in cases of viral infections has been widespread [8–11]. Recent studies suggest, however, that patients around the world are now better informed [12–17].

### The present study

Although instructive, these studies do not tell much regarding the psychological motives that govern patients’ behaviors regarding antibiotics use. As shown in previous studies on patient’s motives for attending or refusing to attend health centers [18] for undergoing/refusing to undergo surgery [19] or for donating organs [20], these psychological motives are likely to form a complex net, and the nature and impact of some of them may be totally unexpected. The goal of the present study was, therefore, to explore, in systematic way, the motives people evoke when they take antibiotics or when they refuse to take them.

## Method

### Participants

The participants were a convenience sample of adults enrolled during daylight hours by two trained research assistants. Participants were approached in different public areas of Toulouse, France (e.g., the campus, post offices, schools, markets). Seven hundred fifty persons were approached, and 418 (56%) agreed to participate. All the participants who agreed to take part in the study had been prescribed antibiotics in the past by their physician. Most of the time, they have decided to take them but sometimes, they have decided not to take them or to discontinue the treatment. All participants provided informed consent. Their demographic characteristics are shown in Table 1.

**Table 1:** Demographic characteristics of the participants. Mean scores observed for each factor as a function of participants’ characteristics

	Mean Factor Score as a Function of Demographic Characteristics										
	Agree to Take						Refuse to Take				
Characteristics	I	II	III	IV	V	VI	I	II	III	IV	*N*
Age											
19–24 Years	8.53	5.07	4.59	4.41	6.77	4.05	3.36	6.61	8.49	5.04	108
25–31 Years	9.88	6.02	5.25	4.75	7.56	4.84	3.22	6.49	8.94	4.91	97
32–47 Years	7.95	6.28	3.65	4.13	6.86	4.55	4.27	6.05	9.13	5.57	111
47 Years +	7.55	5.77	3.30	4.49	6.81	4.63	4.95	6.54	9.03	4.72	98
Gender											
Males	7.32	5.34	4.16	4.30	6.11	4.22	4.54	6.07	9.21	5.01	146
Females	9.08	6.02	4.17	4.48	7.46	4.67	3.62	6.60	8.76	5.13	271
Education											
Primary	7.32	6.03	4.03	4.60	6.06	4.57	4.85	6.45	9.00	5.16	86
Secondary	8.64	5.92	4.02	4.67	6.95	4.52	3.87	6.38	8.96	5.05	219
Tertiary	8.93	5.29	4.54	3.74	7.72	4.43	3.39	6.41	8.75	5.07	113
Children											
No	9.07	5.72	4.64	4.45	7.05	4.52	3.39	6.47	8.83	5.14	227
Yes	7.70	5.84	3.59	4.35	6.88	4.49	4.60	6.32	9.00	5.00	191
Often Ill											
No	8.32	5.69	4.04	4.34	7.03	4.46	3.91	6.29	8.93	4.99	327
Yes	9.01	6.08	4.61	4.68	6.73	4.76	3.93	6.94	8.90	5.34	87
Current Health											
Bad	8.43	6.86	3.55	4.04	6.49	4.35	4.79	6.59	8.94	5.74	44
Good	8.47	5.65	4.24	4.46	7.04	4.55	3.81	6.41	8.94	4.99	370
Number of prescriptions of antibiotics last year											
None	7.87	4.92	3.53	3.94	6.30	4.13	3.42	6.28	9.21	4.74	152
One	8.40	6.01	3.93	4.62	6.85	4.22	4.26	6.48	9.48	5.12	108
Two	9.59	6.76	4.59	4.90	7.95	5.63	4.04	6.40	8.35	5.38	72
More	8.69	6.13	5.24	4.58	7.52	4.63	4.32	6.60	8.16	5.36	84
Out of counter consumption of antibiotics											
Never	8.40	5.84	4.23	4.55	7.08	4.63	3.97	6.57	9.02	5.11	335
Sometimes	8.63	5.50	3.86	3.86	6.58	4.04	3.85	5.64	8.46	4.91	82
Keeps antibiotics for further use											
Never	8.36	5.82	4.25	4.52	7.08	4.68	3.89	6.40	8.86	4.84	300
Sometimes	8.59	5.63	3.91	4.04	6.69	3.95	4.14	6.51	9.03	5.74	114
Uses to stop treatment before completion											
Never	8.34	5.64	3.97	4.10	6.77	4.65	4.12	5.82	8.43	4.60	177
Sometimes	8.55	5.89	4.31	4.64	7.15	4.40	3.76	6.80	9.25	5.40	240
Asks for antibiotics											
Never	8.39	5.65	4.20	4.35	6.85	4.51	3.81	6.40	8.97	5.07	368
Sometimes	9.07	6.79	3.88	4.88	7.99	4.63	4.76	6.57	8.62	5.29	47
Has been forced to change treatment											
Never	8.24	5.41	4.04	4.24	6.62	4.38	3.85	6.26	8.96	4.84	321
Sometimes	9.17	6.99	4.51	4.99	8.21	4.92	4.25	6.88	8.77	5.78	93
Has been the victim of side effects											
Never	8.03	5.43	4.06	4.34	6.59	4.34	3.98	6.02	8.91	4.67	273
Yes	9.23	6.42	4.36	4.54	7.68	4.80	3.87	7.12	8.92	5.86	144
Has experienced useless treatment with antibiotics											
Never	8.45	5.38	4.10	4.17	6.65	4.49	3.82	5.82	8.45	4.58	254
Yes	8.45	6.44	4.28	4.81	7.57	4.55	4.19	7.32	9.67	5.87	161
Thinks that antibiotics have many side effects											
No	8.69	5.55	4.41	4.44	6.82	4.58	4.03	5.83	8.45	4.64	231
Yes	8.43	6.19	3.86	4.54	7.32	4.39	3.88	7.28	9.44	5.63	171
Thinks that antibiotics are generally effective											
No	7.60	6.39	4.13	5.07	6.75	4.83	3.46	7.90	10.00	6.26	35
Yes	8.56	5.71	4.23	4.41	7.05	4.51	4.05	6.12	8.68	4.94	362
Thinks that bacterial resistance is a big public health issue											
No	9.49	5.48	5.10	4.53	6.91	4.72	3.25	4.34	7.12	3.75	84
Yes	8.23	5.94	3.97	4.44	7.14	4.45	4.22	7.04	9.39	5.53	320

Agree to take: I = Appropriate prescription, II = Protective device, III = Enjoyment, IV = Others’ pressure, V = Work imperative, VI = Personal autonomy

Refuse to take: I = Secondary gain, II = Bacterial resistance, III = Self-defense, IV = Lack of trust

### Material

Two separate questionnaires were created (a) a 60-item questionnaire listing reasons for which the person has taken antibiotics in the past, and (b) a 70-item questionnaire listing reasons for which the person has sometimes refused to take antibiotics. A list of 100 items was created on the basis of previous literature on antibiotics consumption [10, 11, 13, 15, 21–23] and on motivation [24, 25]. This list was shown to a focus group of four adults who were members of the public. They reformulated 48 items judged as ambiguous and suggested 22 additional items based on their personal views. This augmented list was then presented to another focus group who suggested 8 additional items.

The common wording of all items – “One of the reasons why I have been led to take (to refuse to take) antibiotics was” – was chosen to reflect the fact that several motives can be operating at the same time or at different times for the same person [25]. A 15-point scale was printed following each sentence. The two extremes of the scales were labeled “Never happened for this motive” (1) and “Frequently happened” (15). The questionnaire is shown in Additional file 1.

### Procedure

Participants answered individually in a quiet room. Half of the participants were presented with the reason-to-take-antibiotics items first and then with the reason-to-refuse items. The other participants were presented with the items in the reverse order. The questionnaires took approximately 50 min to complete. Then, participants were presented with a questionnaire regarding their demographics and personal experience with antibiotics. The research adhered to the legal requirements of the study country: informed consent was obtained and participants’ anonymity was respected.

### Data analyses

Mean scores of the reason-to-take items and of the reason-not-to-take items were computed. Two separate exploratory factor analyses were conducted, one on each set of items. They showed that 24 reason-to-take items and 34 reason-not-to-take items did not load (correlation < .30) on any factor or loaded on more than one factor. They were removed from the analyses, and a second set of factor analyses was conducted. Six interpretable factors (68% of the variance) with eigen-values ranging from 1.11 to 14.43 were observed in the reason-to-take condition, and four interpretable factors (66% of the variance) with eigen-values ranging from 1.45 to 14.88 were observed in the reason-not-to-take condition. Varimax rotations were performed. Ten mean factor scores were computed. A series of forward linear stepwise regression analyses was conducted with the demographic characteristics as the independent variables and the ten scores as the dependent variables.

## Results

One hundred and forty seven males and 271 females aged 18–85 years participated in the study. The mean scores of the reason-to-take items ranged from 2.21 to 9.75 (out of 15). Main results of the first exploratory factor analysis are shown in Table 2.

**Table 2:** Results of the second factor analysis on the agree-to-take items. Means and standard deviations. Cronbach’s alpha. Only four items for each factor – the ones with the highest loadings – are shown

Items	Factors							
One of the reasons why I have been led to take antibiotics was that …	I	II	III	IV	V	VI	*M*	*SD*
... it seemed to be the appropriate treatment	.86	.02	.09	.12	.03	.19	8.22	5.40
... I wished to fight an infection.	.86	.03	.09	.09	−.00	.18	8.46	5.52
... simply because the physician had prescribed them.	.85	−.13	.11	.10	.05	.02	9.44	5.45
... I considered that to take them was reasonable	.84	.14	.06	.13	.01	.22	7.65	5.22
... I particularly feared this kind of infection.	.08	.76	.02	.15	.17	.08	6.66	4.87
... I was not able to put up with the idea that micro-organisms were invading my body.	.10	.67	.06	.28	.14	.20	4.31	4.19
... I wished to reassure and comfort myself.	.03	.65	.19	.23	.27	.03	5.26	4.70
... I wished to quickly recover my place in the family.	−.26	.61	.01	.28	.38	.19	5.46	5.08
... I wished to go out with friends.	.30	.05	.78	.06	.19	.12	4.67	4.44
... I wished to go out in order to change my mind.	.27	.13	.77	.18	.03	.16	3.71	3.95
... I didn’t wish to miss a friendly (or romantic) rendezvous.	.15	.22	.76	.14	.24	.11	4.44	4.27
... I wanted to be able to go to a celebration.	.12	.15	.72	.20	.29	.17	3.82	3.98
... owing to my health state my relatives suggested me to do it.	.18	.31	.22	.81	.14	.10	5.44	4.68
... I was aware that significant persons were preoccupied because of my bad health.	.19	.23	.31	.74	.14	.28	3.78	4.06
... I didn’t want to add anything to people’s concerns about me.	.08	.25	.18	.73	.18	.07	4.13	4.10
... owing to my current state of health my partner strongly insisted I do so.	.25	.08	.18	.73	.02	.26	4.19	4.33
... it was necessary to be in good health for assuming my responsibilities at work (or in my work team)	.06	.25	.15	.05	.73	.25	7.07	5.17
... I wanted to be in good shape because of an important event (to pass an exam or to meet with business partners).	.23	.19	.27	.22	.69	.05	7.85	5.11
... I wanted to complete something important.	−.05	.28	.29	.20	.68	.14	5.80	4.81
... I wanted to be able to go and work or study.	.35	.24	.17	.18	.63	.21	7.18	5.02
... I didn’t want to be a weight for other people.	.29	.20	.12	.22	.21	.71	4.51	4.58
... I didn’t want to depend on other people because of my illness.	.28	.24	.15	.28	.17	.70	4.23	4.46
... I wanted to keep control over certain situations.	.31	.12	.25	.16	.35	.63	4.31	4.40
... I didn’t wish to bother people with my illness.	.35	.16	.19	.30	.11	**.**60	4.98	4.75
Explained variance	7.73	4.06	4.02	3.65	2.99	2.68		
Percentage of variance	.21	.11	.11	.10	.08	.07		
*M*	8.46	5.78	4.17	4.41	6.99	4.51		
*SD*	4.89	3.71	3.60	3.53	4.13	3.86		
Mean score > 8	251	117	77	67	180	75		
Cronbach’s alpha	.93	.79	.89	.89	.84	.87		

I = Appropriate prescription, II = Protective device, III = Enjoyment, IV = Others’ pressure, V = Work imperative, VI = Personal autonomy

The first factor (21% of the variance) was labelled *Appropriate prescription* since it loaded on items expressing the idea that antibiotics were prescribed by a qualified physician and that this prescription looked reasonable to the participants’ eyes. The mean of the four items with the highest loadings was 8.46--*SD* (standard deviation) = 4.89--, the highest value observed. The second factor—*Protective device* (11% of the variance)—expressed the idea that antibiotics can protect the body from bacterial invasion, *M* (mean) = 5.78, *SD* = 3.71. The third factor—*Enjoyment* (11%)—expressed the idea that antibiotics were considered as a quick fix allowing someone to go out and celebrate the week-end as usual (*M* = 4.17, *SD* = 3.60). The fourth factor—*Others’ pressure* (10%)—expressed the idea that antibiotics were taken mainly in order to reassure close relatives (*M* = 4.41, *SD* = 3.53). The fifth factor—*Work imperative* (8%)—expressed the idea that antibiotics were taken mainly to be able to achieve important work (*M* = 6.99, *SD* = 4.13). Finally, the sixth factor—*Personal autonomy* (7%)—expressed the idea that through the taking of antibiotics one can shorten one’s dependence upon others (*M* = 4.51, *SD* = 3.86).

The mean scores of the reason-to-refuse items ranged from 2.29 to 10.61 (out of 15). Main results of the second factor analysis are shown in Table 3. The first factor (34% of the variance) was labelled *Secondary Gain* since it loaded on items expressing the idea that through prolonged illness one can benefit from increased social support and one may also be able to control more easily one’s social environment (*M* = 3.92, *SD* = 4.27). The second factor—*Bacterial Resistance* (14% of the variance)—expressed the idea that the irresponsible use of antibiotics may facilitate the process of bacterial resistance (*M* = 6.41, *SD* = 3.87). The third factor—*Self-defense* (10%)—expressed the idea that the body was able to defend itself against the infection, in particular when it was not severe (*M* = 8.92, *SD* = 3.77). Finally, the fourth factor—*Lack of trust* (8%)—expressed the idea that one may not always be fully confident in the prescriber’s competence (*M* = 5.09, *SD* = 3.61).

**Table 3:** Results of the second factor analysis on the refuse-to-take items. Means and standard deviations. Cronbach’s alpha. Only four items for each factor – the ones with the highest loadings – are shown

Items	Factors					
One of the reasons why I refused to take antibiotics was that … .	I	II	III	IV	*M*	*SD*
... I wished, by prolonging my illness, that people keep being considerate to me.	.92	.04	.13	.05	4.08	4.81
... being ill was an opportunity to have company.	.88	.05	.17	.05	3.74	4.45
... I wished, by being ill, to keep being cared by my relatives.	.88	.04	.11	.11	3.68	4.35
... by keeping being ill, I could obtain important benefits.	.85	.01	.19	.05	4.27	4.83
… the abuse of antibiotics eases the process of bacterial resistance.	.28	.84	.25	.16	7.94	5.21
... the development of bacterial resistance constitutes a threat for future generations.	.06	.81	.18	−.10	5.80	4.78
... I feared that the taking of antibiotics would reduce, in the long term, my natural defenses.	.04	.77	.15	.28	7.38	5.02
... I had learned that irresponsible taking of antibiotics facilitated mutations among bacteria, which consequences were unpredictable.	.08	.73	.03	.19	5.40	4.54
... I thought that my organism was able to defend itself alone.	.12	.34	.80	.28	8.05	4.92
... I considered that medicines were not needed for recovering.	.18	.09	.79	.09	7.79	5.15
... I considered that the illness was not severe enough.	.12	.24	.66	.30	8.75	5.10
... I was not confident in the prescribing physician.	.20	.21	.16	.73	4.75	4.56
... another physician had told me not to take them.	.13	.26	−.00	.64	5.43	4.77
... I disagreed with the physician’s opinion.	.30	.26	.25	.63	5.59	4.61
... in general, I don’t trust physicians.	.32	.28	.23	.62	4.54	4.32
Explained variance	12.14	4.94	3.52	3.03		
Percentage of explained variance	.34	.14	.10	.08		
M	3.94	6.41	8.92	5.09		
SD	4.27	3.87	3.77	3.61		
Mean score > 8	83	138	264	90		
Cronbach’s alpha	.94	.86	.81	.80		

I = Secondary gain, II = Bacterial resistance, III = Self-defense, IV = Lack of trust

Table 1 shows the relationship between participants’ characteristics and scores on each factor of motives and Table 4 shows the results from the stepwise regression analyses. *Appropriate prescription* was significantly associated with gender—ß (beta) = .16--and number of children (ß = −.13). *Protective device* was only associated with change of treatment (ß = .18). *Enjoyment* was associated with age (ß = −.14), number of therapies (ß = .15) and concerns with public health issues (ß = −.10). *Work imperative* was associated with gender (ß = .13), number of antibiotic treatment in the past year (ß = .09) and change of treatment (ß = .13). *Secondary gain* was only associated with age (ß = .17). *Bacterial resistance* was associated with personal experience of inefficacy (ß = .15), conviction that antibiotics are in general useless (ß = .13) and expressed concerns about resistance (ß = .27). *Self-defense* was associated with personal experience of inefficacy (ß = .13) and expressed concerns about resistance. Finally, *Lack of trust* was similarly associated with personal experience of inefficacy (ß = .15) and expressed concerns about resistance (ß = .18).

**Table 4:** Main Results From the Stepwise Linear Regression Analyses

Criterion	Predictors	β	*t*	*F*	*p*	R
Appropriate Prescription				9.99	.001	.21
	Gender	.16	3.40		.001	
	Number of children	−.13	2.67		.01	
Protective Device				13.48	.001	.18
	Change of Treatment	.18	3.67		.001	
Enjoyment				8.99	.001	.25
	Number of Prescriptions	.15	2.99		.003	
	Age	−.14	2.92		.003	
	Resistance	−.10	2.10		.04	
Work Imperative				7.64	.001	.23
	Change of Treatment	.13	2.52		.02	
	Gender	.13	2.75		.005	
	Number of Prescriptions	.09	1.71		.09	
Secondary Gains				12.99	.001	.17
	Age	.17	3.60		.001	
Bacterial Resistance				18.30	.001	.34
	Resistance	.27	5.69		.001	
	Inefficacy	.14	3.00		.001	
	Generally Useless	−.13	2.78		.01	
Self-Defense				7.53	.001	.27
	Resistance	.22	4.67		.001	
	Inefficacy	.13	2.68		.01	
Lack of Trust				13.42	.001	.25
	Resistance	.18	3.67		.001	
	Inefficacy	.15	3.13		.001	

## Discussion

The most strongly endorsed motive to agree to take antibiotics, especially among females and patients with children, was that they had been prescribed by a physician. This reason was, however, associated with the idea that the physician’s prescription was at the same time judged appropriate and reasonable. This means that, if in general people were willing to follow the prescriber’s recommendations, they were unwilling to do so blindly.

The second most strongly endorsed motive, especially among females, and among people who had experienced trouble with antibiotics in the past, was work pressure. In addition, the persons invoking this kind of motive tended more than others to take antibiotics on a regular basis. Antibiotics may thus be viewed by some people as a way to enhance performance at work.

The third most strongly endorsed motive, especially among people who had experienced trouble with antibiotics, was related to the fear and suffering engendered by the infection. It is logical that people in bad health who have experienced unsuccessful treatment are willing to take, more than others (and at times to ask for), antibiotics to protect themselves; that is, to keep themselves able to be well and to perform well in their environment.

Three other kinds of motives were also found, although they were less strongly endorsed than the others: close relatives’ concerns, personal autonomy, and enjoyment. The first two motives were related to family and social life but the third one is more concerning. People who more frequently than others endorsed enjoyment-type motives tended to discount the severity of bacterial resistance. They were younger and reported having taken antibiotics more than twice the past year. This suggests that a small, but not negligible segment of the sample (18%) considers that antibiotics are just consumption goods that can be freely used.

The most strongly endorsed motive to refuse to take antibiotics was that one’s body was seen as not severely endangered by the infection and, as a result, would be able to defend itself successfully. This result was consistent with findings by Jonsson and Haraldsson [26]. This kind of motive was endorsed especially by people who, more than others thought that antibiotics are ineffective.

The second most strongly endorsed motive to refuse was directly related to concern about bacterial resistance. This result was consistent with findings by Finkelstein et al. [23], but this commendable vision seemed to have its limitations: it was expressed especially by people who (a) had experienced troubles with treatment with antibiotics, (b) thought that antibiotics are generally ineffective, and (c) did not hesitate to stop treatment inappropriately.

Two other motives to refuse antibiotics were found: (a) the presence of secondary gain associated with prolonged illness, especially among older people, and (b) lack of trust in the prescriber, especially among people who had had troubles with past treatment with antibiotics and at the same time reported behaving in a way that is paradoxical because it was potentially dangerous for themselves (i.e., keeping antibiotics after treatment for later use).

### Limitations

The study has at least two limitations. First, motives were assessed through self-reports. Participants’ responses were, however, clearly structured: If they had consciously decided to misreport their motives, responses would have been given in a more or less random way and, as a result, no clear factor structure could have been found. Now that the complete structure of motives is known, it will be possible, in future studies, conducted in collaboration with physicians, to contact people who have recently been prescribed antibiotics, to ask them whether they have taken these antibiotics, and, using a shortened six-item (or four-item) version of the questionnaire, to assess the reasons why they have taken them (or not taken them or discontinued the treatment). Second, the sample was conducted in a single site in France. Its results must, therefore, be generalized with care to other populations in the country, namely to those who live in rural settings. In addition, the two models of motives have been issued from exploratory factor analyses. They need to be confirmed on other samples, using confirmatory factor analysis, and measurement invariance has to be assessed (men vs. women, young vs. aged, often sick or not).

## Conclusions

People are generally willing to follow their physician’s prescription of antibiotics. In our study, however, *Appropriate prescription*, although the leading motive, was not rated as highly as could have been expected: its mean rating was located only slightly above the center of the response scale. This implies that people would be willing to take antibiotics if instructed to do so and at the same time, for example: (a) told that antibiotics will attenuate their physical suffering or (b) reassured that, owing to taking antibiotics, they will be able more quickly to achieve an important task.

Although most people seemed to be aware that bacterial resistance was a big public health issue, a minority (about 21%) did not agree with this view, and, what is more concerning, they were mostly among those who reported that, when they are ill, they do not hesitate to use antibiotics simply in order to go out and have fun with friends. It should be explained to these people that when ill, the best they could do is to stay at home and try not to contaminate large groups of people.

Although it is certainly a good thing that people sometimes are unwilling to take antibiotics, there seemed to be a gap between the wisdom or altruism of their reasons and what they reported regarding their behavior. They were aware more than others of the public health issue and also of their body’s capacity to defend itself against infections, but at the same time they also tended more than others to report behaviors that were at variance with their motives. In particular they did not hesitate to stop treatment before it had been completed; that is, to do what would facilitate mutations and adaptations in microorganisms. In fact, they seemed to be essentially acting out of previous negative personal experience with antibiotics because their statements regarding bacterial resistance as a big public health issue were more rhetorical than grounded in even minimal understanding (see also Napolitano et al. [13]). In other words, even people who express a willingness to take antibiotics only if really needed must be educated about the mechanisms by which micro-organisms adapt to human defenses [27].

Finally, one out of five participants expressed lack of trust in physicians and treatment with antibiotics. Unfortunately, these people, more frequently than others, reported behaviors that were potentially more dangerous to themselves (e.g., shortening duration of treatment) than anything physicians could recommend or prescribe in these circumstances.

Overall, a notable proportion of people report adopting behaviors that are more beneficial to micro-organisms than to humans and other animals; that is, behaviors that are likely to increase bacterial resistance. If taught that bacterial resistance is a planetary health concern, these people would certainly not be surprised, and a huge majority would agree. They would even be likely to report behaviors such as shortening the duration of treatment and using past-prescribed antibiotics as proofs of their good intentions. As a result, they must be taught that their behavior is counterproductive, and that their small actions have global consequences.

In summary, in each instance of consultation involving prescription of antibiotics--and particularly if physicians have detected erroneous beliefs, physicians must remind patients that antibiotics help fight dangerous micro-organisms, that temporary isolation is often the best way to limit contagion, that stopping treatment before completion is exactly what helps micro-organisms to become stronger, and that inappropriate action by a minority can affect the whole human population. This information must, however, not be delivered in a confrontational way: As stressed by the promoters of motivational interviewing [28], motivation to act in a determined way can only be elicited from the patient; it cannot be imposed from outside.

## Additional file

Additional file 1: Questionnaire. (PDF 305 kb)

## Abbreviations

BRICS: Brazil, Russia, India, China and South-Africa
M: Mean
SD: Standard deviation
ß: Beta
