# Supplementary material for: Why patients want to take or refuse to take antibiotics: an inventory of motives
Source: BMC Public Health. 2019 Apr 27;19:441. doi: 10.1186/s12889-019-6834-x (PMC6487028; doi:10.1186/s12889-019-6834-x)
Supplement: Supplementary file 1 — Questionnaire. (PDF 305 kb) [file 12889_2019_6834_MOESM1_ESM.pdf]

Questionnaire

Reasons for taking (or not taking) antibiotics

One of the reasons why I have been led to take antibiotics was that it seemed to be the appropriate treatment.

Never happened for this motive 1 2 3 4 5 6 7 8 9 10 11 12 13 14 15 *Frequently happened*

One of the reasons why I have been led to take antibiotics was that I wished to fight an infection.

Never happened for this motive 1 2 3 4 5 6 7 8 9 10 11 12 13 14 15 *Frequently happened*

One of the reasons why I have been led to take antibiotics was simply because the physician had prescribed them.

Never happened for this motive 1 2 3 4 5 6 7 8 9 10 11 12 13 14 15 *Frequently happened*

One of the reasons why I have been led to take antibiotics was that I considered that to take them was reasonable.

Never happened for this motive 1 2 3 4 5 6 7 8 9 10 11 12 13 14 15 *Frequently happened*

One of the reasons why I have been led to take antibiotics was that I particularly feared this kind of infection.

Never happened for this motive 1 2 3 4 5 6 7 8 9 10 11 12 13 14 15 *Frequently happened*

One of the reasons why I have been led to take antibiotics was that I was not able to put up with the idea that micro-organisms were invading my body.

Never happened for this motive 1 2 3 4 5 6 7 8 9 10 11 12 13 14 15 *Frequently happened*

One of the reasons why I have been led to take antibiotics was that I wished to reassure and comfort myself.

Never happened for this motive 1 2 3 4 5 6 7 8 9 10 11 12 13 14 15 *Frequently happened*

One of the reasons why I have been led to take antibiotics was that I wished to quickly recover my place in the family.

Never happened for this motive 1 2 3 4 5 6 7 8 9 10 11 12 13 14 15 *Frequently happened*

One of the reasons why I have been led to take antibiotics was that I wished to go out with friends.

Never happened for this motive 1 2 3 4 5 6 7 8 9 10 11 12 13 14 15 *Frequently happened*

One of the reasons why I have been led to take antibiotics was that I wished to go out in order to change my mind.

Never happened for this motive 1 2 3 4 5 6 7 8 9 10 11 12 13 14 15 *Frequently happened*

One of the reasons why I have been led to take antibiotics was that I didn’t wish to miss a friendly (or romantic) rendezvous.

Never happened for this motive 1 2 3 4 5 6 7 8 9 10 11 12 13 14 15 *Frequently happened*

One of the reasons why I have been led to take antibiotics was that I wanted to be able to go to a celebration.

Never happened for this motive 1 2 3 4 5 6 7 8 9 10 11 12 13 14 15 *Frequently happened*

One of the reasons why I have been led to take antibiotics was that owing to my health state my relatives suggested me to do it.

Never happened for this motive 1 2 3 4 5 6 7 8 9 10 11 12 13 14 15 *Frequently happened*

One of the reasons why I have been led to take antibiotics was that I was aware that significant persons were preoccupied because of my bad health.

Never happened for this motive 1 2 3 4 5 6 7 8 9 10 11 12 13 14 15 *Frequently happened*

One of the reasons why I have been led to take antibiotics was that I didn't want to add anything to people's concerns about me.

Never happened for this motive 1 2 3 4 5 6 7 8 9 10 11 12 13 14 15 *Frequently happened*

One of the reasons why I have been led to take antibiotics was that owing to my current state of health my partner strongly insisted I do so.

Never happened for this motive 1 2 3 4 5 6 7 8 9 10 11 12 13 14 15 *Frequently happened*

One of the reasons why I have been led to take antibiotics was that it was necessary to be in good health for assuming my responsibilities at work (or in my work team)

Never happened for this motive 1 2 3 4 5 6 7 8 9 10 11 12 13 14 15 *Frequently happened*

One of the reasons why I have been led to take antibiotics was that I wanted to be in good shape because of an important event (to pass an exam or to meet with business partners).

Never happened for this motive 1 2 3 4 5 6 7 8 9 10 11 12 13 14 15 *Frequently happened*

One of the reasons why I have been led to take antibiotics was that I wanted to complete something important.

Never happened for this motive 1 2 3 4 5 6 7 8 9 10 11 12 13 14 15 *Frequently happened*

One of the reasons why I have been led to take antibiotics was that I wanted to be able to go and work or study.

Never happened for this motive 1 2 3 4 5 6 7 8 9 10 11 12 13 14 15 *Frequently happened*

One of the reasons why I have been led to take antibiotics was that I didn't want to be a weight for other people.

Never happened for this motive 1 2 3 4 5 6 7 8 9 10 11 12 13 14 15 *Frequently happened*

One of the reasons why I have been led to take antibiotics was that I didn't want to depend on other people because of my illness.

Never happened for this motive 1 2 3 4 5 6 7 8 9 10 11 12 13 14 15 *Frequently happened*

One of the reasons why I have been led to take antibiotics was that I wanted to keep control over certain situations.

Never happened for this motive 1 2 3 4 5 6 7 8 9 10 11 12 13 14 15 *Frequently happened*

One of the reasons why I have been led to take antibiotics was that I didn't wish to bother people with my illness.

Never happened for this motive 1 2 3 4 5 6 7 8 9 10 11 12 13 14 15 *Frequently happened*

One of the reasons why I refused to take antibiotics was that I wished, by prolonging my illness, that people keep being considerate to me.

Never happened for this motive 1 2 3 4 5 6 7 8 9 10 11 12 13 14 15 *Frequently happened*

One of the reasons why I refused to take antibiotics was that being ill was an opportunity to have company.

Never happened for this motive 1 2 3 4 5 6 7 8 9 10 11 12 13 14 15 *Frequently happened*

One of the reasons why I refused to take antibiotics was that I wished, by being ill, to keep being cared by my relatives.

Never happened for this motive 1 2 3 4 5 6 7 8 9 10 11 12 13 14 15 *Frequently happened*

One of the reasons why I refused to take antibiotics was that by keeping being ill, I could obtain important benefits.

Never happened for this motive 1 2 3 4 5 6 7 8 9 10 11 12 13 14 15 *Frequently happened*

One of the reasons why I refused to take antibiotics was that the abuse of antibiotics eases the process of bacterial resistance.

Never happened for this motive 1 2 3 4 5 6 7 8 9 10 11 12 13 14 15 *Frequently happened*

One of the reasons why I refused to take antibiotics was that the development of bacterial resistance constitutes a threat for future generations.

Never happened for this motive 1 2 3 4 5 6 7 8 9 10 11 12 13 14 15 *Frequently happened*

One of the reasons why I refused to take antibiotics was that I feared that the taking of antibiotics would reduce, in the long term, my natural defenses.

Never happened for this motive 1 2 3 4 5 6 7 8 9 10 11 12 13 14 15 *Frequently happened*

One of the reasons why I refused to take antibiotics was that I had learned that irresponsible taking of antibiotics facilitated mutations among bacteria, which consequences were unpredictable.

Never happened for this motive 1 2 3 4 5 6 7 8 9 10 11 12 13 14 15 *Frequently happened*

One of the reasons why I refused to take antibiotics was that I thought that my organism was able to defend itself alone.

Never happened for this motive 1 2 3 4 5 6 7 8 9 10 11 12 13 14 15 *Frequently happened*

One of the reasons why I refused to take antibiotics was that I considered that medicines were not needed for recovering.

Never happened for this motive 1 2 3 4 5 6 7 8 9 10 11 12 13 14 15 *Frequently happened*

One of the reasons why I refused to take antibiotics was that I considered that the illness was not severe enough.

Never happened for this motive 1 2 3 4 5 6 7 8 9 10 11 12 13 14 15 *Frequently happened*

One of the reasons why I refused to take antibiotics was that I was not confident in the prescribing physician.

Never happened for this motive 1 2 3 4 5 6 7 8 9 10 11 12 13 14 15 *Frequently happened*

One of the reasons why I refused to take antibiotics was that another physician had told me not to take them.

Never happened for this motive 1 2 3 4 5 6 7 8 9 10 11 12 13 14 15 *Frequently happened*

One of the reasons why I refused to take antibiotics was that I disagreed with the physician's opinion.

Never happened for this motive 1 2 3 4 5 6 7 8 9 10 11 12 13 14 15 *Frequently happened*

One of the reasons why I refused to take antibiotics was that in general, I don't trust physicians.

Never happened for this motive 1 2 3 4 5 6 7 8 9 10 11 12 13 14 15 *Frequently happened*

Age: ……………….

Gender: Male ☐ Female ☐

Highest level of school completed: Primary ☐ Secondary ☐ Tertiary ☐

Do you have children? No ☐ Yes ☐

Are you often ill? No ☐ Yes ☐

How is your current health? Bad ☐ Good ☐

How many prescriptions of antibiotics did you get last year?

None ☐ One ☐ Two ☐ More ☐

Have you ever used over-the-counter antibiotics? Never ☐ Sometimes ☐

Have you ever kept antibiotics for further use? Never ☐ Sometimes ☐

Have you ever stopped treatment before completion?

Never ☐ Sometimes ☐

Have you ever asked for antibiotics? Never ☐ Sometimes ☐

Have you ever been forced to change treatment? Never ☐ Sometimes ☐

Have you ever been victim of side effects? Never ☐ Yes ☐

Have you ever experienced useless treatment with antibiotics? Never ☐ Yes ☐

Do antibiotics have side effects? No ☐ Yes ☐

Are antibiotics generally effective? No ☐ Yes ☐

Is bacterial resistance a big public health issue: No ☐ Yes ☐
